# An open-source GIS-enabled lookup service for Nagoya Protocol party information

**DOI:** 10.1093/database/baaa014

**Published:** 2020-04-11

**Authors:** Hendrikje Seifert, Marc Weber, Frank Oliver Glöckner, Ivaylo Kostadinov

**Affiliations:** 1 Max-Planck Institute for Marine Microbiology, Celsiusstraße 1, 28359 Bremen, Germany; 2 Life Sciences and Chemistry, Campus Ring 1, 28759 Bremen, Germany; 3 Computing Center, Helmholtz Center for Polar and Marine Research, Am Handelshafen 12, 27570 Bremerhaven, Germany; 4 GFBio—Gesellschaft für Biologische Daten e.V., Campus Ring 1, 28759 Bremen, Germany; 5 University of Bremen, MARUM, Bibliothekstraße 1, 28359 Bremen

## Abstract

The Nagoya Protocol on Access and Benefit Sharing is a transparent legal framework, which governs the access to genetic resources and the fair and equitable sharing of benefits arising from their utilization. Complying with the Nagoya regulations ensures legal use and re-use of data from genetic resources. Providing detailed provenance information and clear re-usage conditions plays a key role in ensuring the re-usability of research data according to the FAIR (findable, accessible, interoperable and re-usable) Guiding Principles for scientific data management and stewardship. Even with the framework provided by the ABS (access and benefit sharing) Clearing House and the support of the National Focal Points, establishing a direct link between the research data from genetic resources and the relevant Nagoya information remains a challenge. This is particularly true for re-using publicly available data. The Nagoya Lookup Service was developed for stakeholders in biological sciences with the aim at facilitating the legal and FAIR data management, specifically for data publication and re-use. The service provides up-to-date information on the Nagoya party status for a geolocation provided by GPS coordinates, directing the user to the relevant local authorities for further information. It integrates open data from the ABS Clearing House, Marine Regions, GeoNames and Wikidata. The service is accessible through a REST API and a user-friendly web form. Stakeholders include data librarians, data brokers, scientists and data archivists who may use this service before, during and after data acquisition or publication to check whether legal documents need to be prepared, considered or verified. The service allows researchers to estimate whether genetic data they plan to produce or re-use might fall under Nagoya regulations or not, within the limits of the technology and without constituting legal advice. It is implemented using portable Docker containers and can easily be deployed locally or on a cloud infrastructure. The source code for building the service is available under an open-source license on GitHub, with a functional image on Docker Hub and can be used by anyone free of charge.

## Introduction

Currently, an enormous amount of data are being produced as part of environmental and biological research areas like genomics, physiology or biodiversity studies (1). It is challenging to integrate this information and generate knowledge from it because the majority of biological and environmental data is complex, heterogeneous and scattered across various storage platforms and repositories. Problems arising include the lack of information about the provenance and verifiability of data points in terms of their acquisition conditions. Even when sample materials were acquired according to internationally accepted legal standards, integrating the data requires elaborate efforts in data transformation and harmonization. According to the FAIR principles for data stewardship (2), the scattered storage of information impairs the accessibility and re-usability of environmental and biological data. Especially the absence of clear usage licenses complicates the re-usability of scientific data sets. That is why the re-usability principle poses specific requirements on the transparency and compliance with any legal restrictions that apply when sampling under national jurisdiction. Consequently, metadata providing the exact acquisition location is crucial. The `Nagoya Protocol on Access to Genetic Resources and the Fair and Equitable Sharing of Benefits Arising from their Utilization (ABS)’ (3) is an internationally legal framework governing the acquisition and (re-) use of genetic resources as well as the equitable sharing of benefits arising from their utilization. By ensuring a transparent, legally binding environment for the signatories, the framework encourages sustaining and conserving biodiversity by aiming toward clear data handling conditions between research partners. This is important because it means distinct assurance for all parties involved in terms of access to resources, e.g. sampling activities, and it also means agreed benefits to the providing country if resources are eventually taken abroad. The Nagoya Protocol entered into force on 12 October 2014 and as of July 2019 there are 117 signatory countries (parties). With the Nagoya Protocol in place, the provenance of data points is crucial for legally compliant re-use and planning research activities abroad has an extra level of complexity. However, linking publicly available data generated from genetic resources to the required Nagoya-relevant paperwork or even deciding whether Nagoya actually applies is still challenging. Even if a country has not signed the Nagoya Protocol, there may still be general ABS regulations to be considered. That means, once research participants have met the applicable due diligence requirements, the country’s publishing authority provides the unity of those legal documents (prior informed consent, material transfer agreement, etc.) as an Internationally Recognized Certificate of Compliance (IRCC) to the Access and Benefit Sharing Clearing House, which maintains the platform for these records. The published IRCC, or equivalent, will contain general information, the original copy of the permits and the actual subject matter. These records can be found on the ABS Clearing House (ABSCH) country profiles and may include a link to the corresponding data sets, if available. Before sampling, a scientist may not even be aware of where to look for further information or who to contact about details to even establish the relevant documents, or the corresponding geographical information of the sampling location may not be available for all data points.

The National Centre for Biotechnology Information, the European Nucleotide Archive (ENA) and the DNA Databank of Japan host information like genomic context, gene descriptions, transcripts, sequences, taxonomy, DOIs and further metadata containing publication date and comments. These archives report an evidently rapid growth of sequence and genome data by submissions during the past years (4, 5). With such exponential increase of data points in public archives comes the consideration of how to ensure long-term re-usability of all the valuable work put into the data acquisition. Among the information attached to a data sample entry, there may be a geolocation where the sample was retrieved. Following a query on ENA of all the data entries submitted after 12 October 2014 ~47% carry geolocations (as of 2018 February). This coordinate pair can be a starting point to look up the originating country (if not international waters). Having coordinates and hence knowing information on the acquisition country may be the basis for looking up country-specific details and conditions for re-using the data.

The Nagoya Lookup Service enables stakeholders like life scientists, data brokers or data archive administrators to determine whether the Nagoya Protocol applies to a given geographic location, without constituting legal advice. The service can be used at all stages of the data lifecycle: (i) before data generation in order to determine the local authorities to be contacted, in preparation of a planned cruise, when documents need to be prepared, (ii) during data acquisition, when the provenance of the data sets are checked, and (iii) after data publication, before data re-use to verify the signatory status of a country. The lookup service does not only raise awareness but also points users in the right direction in terms of authorities to contact for further details or country-specific agreements and helps linking the genetic resources to the Nagoya framework.

**Figure 1:**
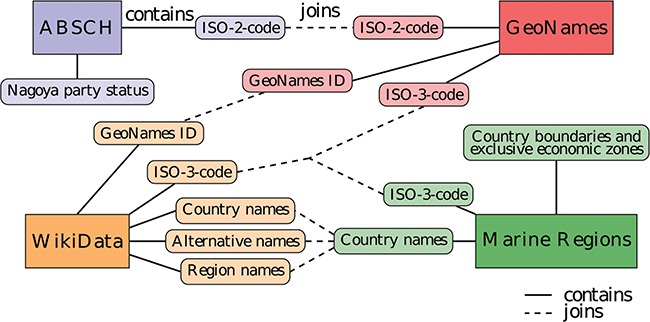
Diagram of the data sources and entity relationships with integrative joins between the different data sources. The same color denotes information originating from the same data source.

## Service development

### Data sources and integration

The following data sources were integrated to establish the lookup service:

- Nagoya Protocol party information from the ABSCH (https://absch.cbd.int/countries), automated access via the ABSCH web service API, https://api.cbd.int/api/v2013/countries. Details include ISO alpha-2 country code and, if applicable, the date a country signed the protocol.
- Geospatial polygons of country borders and exclusive economic zones (EEZs) from Marine Regions (www.marineregions.org), which is managed by the Flanders Marine Institute, accessible via download (not automated because of registration requirement). Details include country names, ISO alpha-3 codes, and polygons representing the combined national borders and EEZ boundaries per country.
- A list of ISO 3166-1 codes from GeoNames (www.geonames.com), accessible via automated file download. The information includes GeoNames IDs (unique GeoNames-specific identifiers), ISO alpha-2 and ISO alpha-3 country codes (abbreviated as ISO-2 and ISO-3 codes in this manuscript). These ISO-2 and ISO-3 country codes are internationally standardized two- and three-letter codes of countries and are included together with dependent territories and special areas of geographical interest, which aid the information integration between various data sources.
- Supplementary country information, including GeoNames IDs, ISO-3 codes, country names, alternative country names and region names from Wikidata (www.wikidata.org), automated access via the SPARQL API.

The lookup service integrates several different data sources in a Postgres relational database (The PostgreSQL Global Development Group, PostgreSQL 10, version 2.0, available at www.postgresql.org) with a PostGIS extension (PostGIS Project, version 2.4, available at www.postgis.net) for handling polygon data and spatial queries. The resulting entity relationship (Figure 1) is the basis of the service. The different parts are predominantly linked by ISO codes, which are standardized for every country. Some links are established by country name. However, a few country names are either spelled differently across the various data sources or may have external territories or names they are alternatively known by (e.g. Ivory Coast a.k.a. Côte d’Ivoire). To overcome this limitation, the database is set up to integrate the data using any of the available shared properties, whichever matches first, being one of the following: ISO codes, country names, external territory/region and alternative names.

### Implementation

The service is implemented using PostgreSQL/PostGIS for the backend. The middleware is programmed in Python (Python Software Foundation, version 3.6.5, available at www.python.org), using the Django Web Framework (Django Software Foundation, available at https://www.djangoproject.com) with cookiecutter-django (available at https://github.com/pydanny/cookiecutter-django) as the basis. The REST API is realized with the Django Rest Framework (available at https://www.django-rest-framework.org). The service is packaged with Docker (Docker Inc., Docker Toolbox for Windows, version 17.07.0, available at https://www.docker.com), including an https-enabled web server (Caddy) and a cache server (Redis), and can be started with a single docker compose command. Thus, the entire service is portable and can easily be deployed both locally and on a cloud infrastructure. The database container is set up to fetch the latest data from all sources, which allow automated access (see 2.1 Data sources and integration), allowing an update to be performed by simply re-starting the container. In order to offer the integration of these functionalities to other systems, programmatic access is given by an API. A user-friendly HTML-based web form is also included.

**Figure 2:**
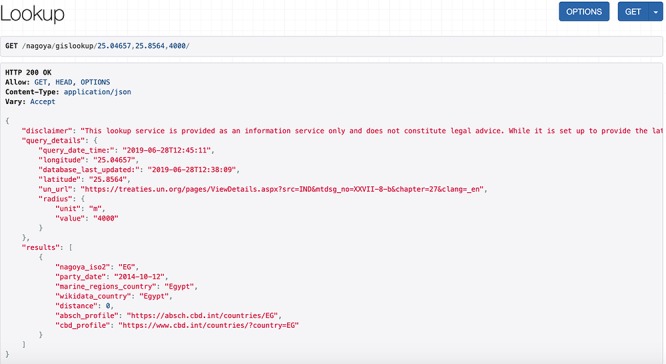
Screenshot of an API request and its JSON response for a coordinate pair and a query radius of 4000 m.

**Figure 3:**
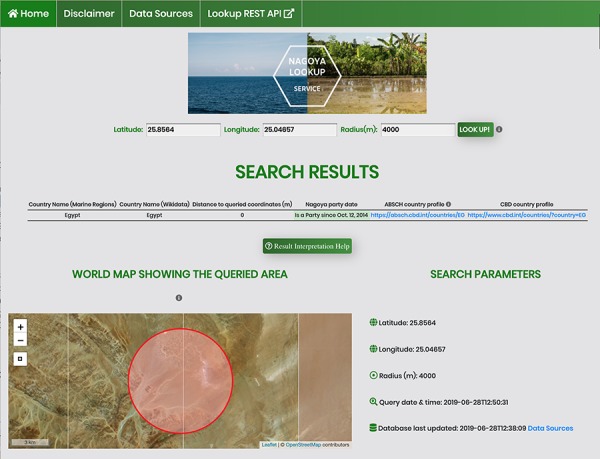
Screenshot of an API request using the HTML form for a coordinate pair and a query radius of 4000 m.

### The lookup service

The service accepts a geographic location as a coordinate pair (decimal notation in WGS84) and an optional query radius in meters, with a default currently set to 5000 m. The output is a list of countries sorted by the distance between the queried coordinate pair and the borders and/or EEZs within the query radius. The output details include the Nagoya party status for each identified country and for signatories it shows external links to the ABSCH country profile and to the Convention on Biological Diversity (CBD) profile. These external websites provide contact information of the National Focal Point (NFP) and Competent National Authority (CNA) of that country, who can be contacted for further information and validation of country-specific details like special agreements. Regardless of whether there are identified countries within the search radius (as opposed to international waters), along with the country list the service always returns the request details, including input coordinates and variables, a disclaimer and a time stamp of the last database update as well as the query time and date and a link to the United Nations (UN) Treaty Collection. This Treaty Collection gathers a general list of Nagoya parties together with a short listing of specific agreements for some countries. This is particularly useful in case there are uncertainties, for example when the service returns a country list different to what the user expected. An exemplary API request (/nagoya/gislookup/25.04657,25.8564,4000/) with its JSON response is displayed in Figure 2 and the equivalent call using the HTML form (/nagoya/lookup/?latitude=25.8564&longitude=25.04657&radius=4000) is shown in Figure 3. Besides the list of results, both include the original query parameters, the timestamp of the request, the timestamp of the last database update and a disclaimer.

## Discussion and outlook

### Conclusion

This innovative lookup service can be used by various stakeholders when planning their experiments, while conducting their research and when publishing their data to estimate the applicability of the Nagoya Protocol to a given location. Thus, it enhances the long-term re-usability of data and contributing to the FAIRness of genetic research material. The Nagoya Lookup Service helps in data management planning even before the sampling has started, as well as complying with legal regulations while processing and re-using data. These possibilities extend to human genomics, as there seems no comparable open-access service for that research field.

### Limitations

Last but not least, since this service does not constitute legal advice, whenever the service is applied, the next step must always be contacting the NFP or other relevant authority indicated on the ABS country profile. To make sure users are aware of this at any time, a disclaimer on the query page states `This lookup service is provided as an information service only and does not constitute legal advice. It is advised to do further legal research on the country related to your search and to contact the National Focal Point and/or Competent National Authority in any case. While this service is set up to provide the latest Nagoya party information, this service relies on third party data sources. This is particularly true with regard to the information and documents on each individual country’s national law which this database does not comprehensively cover. This service additionally contains links to external websites and content originating from third parties. Such external links are not investigated, monitored or checked for accuracy, validity, reliability, availability and completeness by us. Your use of this service and your reliance on any kind of information provided here is solely at your own risk.’

Despite extensive integration work, the service also has limitations due to discrepancies in the open data sources used. For example, some countries have complex relationships and specific legal agreements with their external regions and/or dependent territories resulting in varying Nagoya Protocol signatory statuses for those areas.

In addition, certain geographical regions are claimed or administered by more than one country, which complicates associating those regions to a distinct Nagoya country profile. At the same time, different third-party data sources may provide mismatching, sometimes even contradictory, information ranging from country name spellings to legislative statuses and authoritative aspects. Therefore, the NFPs and the ABSCH remain the principal entities to contact for authoritative information.

At the moment, only three of the four data sources can be retrieved via programmatic access (see section 2.1 Data sources and integration). A regular, automated update for these three sources could be implemented; however, the data from Marine Regions still need to be downloaded manually. The service can potentially be extended to allow users to query a list of coordinate pairs on the website. For extensive use multiple calling of the REST API service is recommended as a simple workaround. Furthermore, the service is currently only available in English; additional languages and other output formats (e.g. comma-separated value) may be offered in the future. Last but not least, given the appropriate data resources, the service can also be extended to cover other relevant legal frameworks, for example additional CBD Convention Protocols.

## Availability and requirements

**Operating Systems:** UNIX-based systems; any operating system with Docker support.

**Availability:** the source code and documentation are available in the GitHub repository: https://github.com/hseifert/nagoya-lookup-service.

This article refers to version 1.0 for which the Docker image is available from https://hub.docker.com/r/hseifert/nagoya-lookup-service.

**License:** MIT (see LICENSE.md file)

**Requirements:** There are some installations and third-party libraries required to start the service, they can be found in the corresponding README.
